# Taxonomic description of a new freshwater *Gammarus* (Crustacea, Amphipoda, Gammaridae) restricted to the upper Hantan River, central Korean Peninsula

**DOI:** 10.3897/BDJ.14.e179349

**Published:** 2026-01-26

**Authors:** Yong-Uk Ahn, Gi-Sik Min

**Affiliations:** 1 Inha University, Incheon, Republic of Korea Inha University Incheon Republic of Korea

**Keywords:** 28S, COI, freshwater, gammarid, Korea, morphology, taxonomy

## Abstract

**Background:**

*Gammarus* Fabricius, 1775 is the largest genus in the family Gammaridae and most species inhabit freshwater and exhibit high regional endemism. To date, 12 freshwater *Gammarus* species are known from South Korea. However, considering the complex topography of the Korean Peninsula, the species diversity of freshwater *Gammarus* may be underestimated.

**New information:**

A new species of the genus *Gammarus* Fabricius, 1775 is described, originating from the Hantan River region, South Korea. *Gammarus kyungsooki* **sp. nov.** is morphologically characterised by the absence of calceoli on antenna 2, the presence of 6–7 clusters of long setae on the posterior margin of peduncular article 4 in antenna 2, long straight setae along the posterior margins of merus to carpus in pereopod 3, a 0–2–0 dorsal spine formula on urosomite 1, plumose setae on the outer margin of the outer ramus of uropod 3 and three or more clusters of setae on the outer margin of each telson lobe. Genetic distance analyses, based on mitochondrial cytochrome *c* oxidase subunit I (COI) and nuclear large-subunit ribosomal RNA (28S) gene sequences, support the distinctiveness of the new species, with interspecific divergences of 18.8%–25.2% (COI) and 1.7%–5.1% (28S).

## Introduction

The genus *Gammarus* Fabricius, 1775 comprises 297 valid species, making it one of the largest genera of Amphipoda (Horton et al. 2025). Members of the genus are distributed across fresh, brackish and marine waters of the Northern Hemisphere and approximately 80% of them inhabit freshwater (Väinölä et al. 2008). Most freshwater *Gammarus* species are geographically restricted and occur as endemic species in local regions, largely because of their limited dispersal ability (Hou et al. 2014, Mamos et al. 2016).

Freshwater ecosystems of the Korean Peninsula, shaped by a complex geological history, exhibit high levels of endemism, with more than 70% of the region covered by mountains and hills containing well-developed streams and rivers that provide isolated habitats for many endemic freshwater organisms (Yoon et al. 2018, Jeon et al. 2022, Lee 2025). The Hantan River in central Korea comprises a complex volcano-fluvial landscape formed during the Quaternary and is notable for high geodiversity (Ryu et al. 2011, Kil et al. 2018). Hu et al. (2021) conducted phylogenetic analyses and suggested that the uplift of the Changbai Mountains in the northern Korean Peninsula during the Miocene played a significant role in the diversification and dispersal of freshwater Gammarus in Northeast Asia and further indicated that the diversity of Korean freshwater *Gammarus* is greater than currently recognised.

Here, we describe a new freshwater amphipod species, *Gammarus kyungsooki* sp. nov., collected from mountain streams in the Hantan River region, South Korea, based on morphological characteristics and genetic distance analyses of the mitochondrial cytochrome *c* oxidase subunit I (COI) and nuclear large-subunit ribosomal RNA (28S) genes.

## Materials and methods

### Sampling and morphological observations

**Figure 1.**
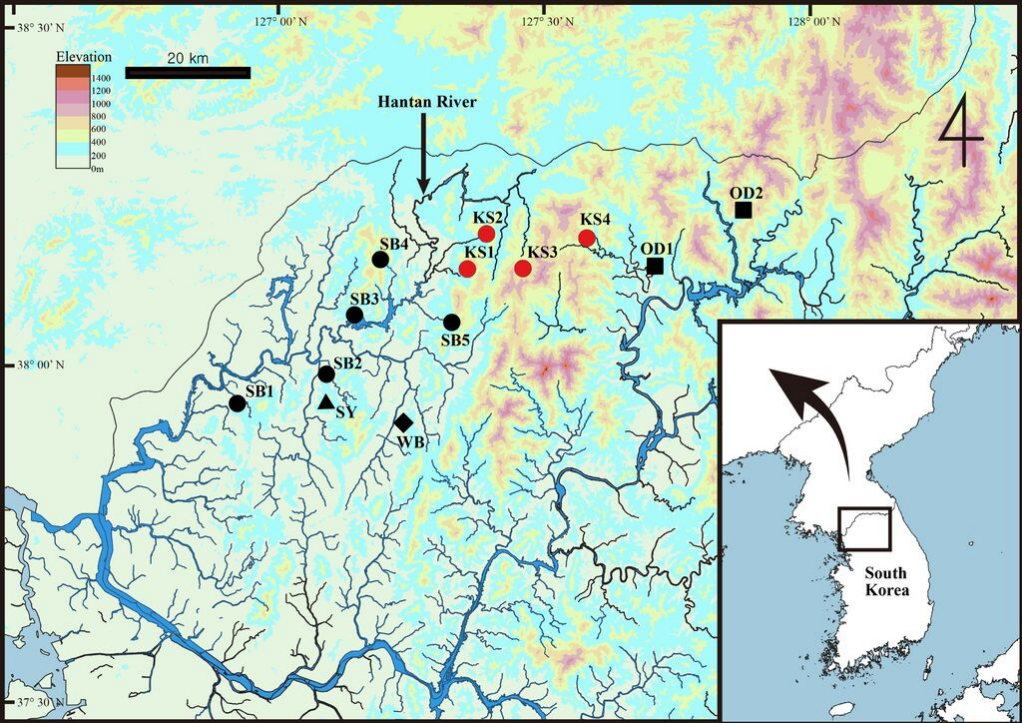
Sampling sites of *Gammarus* specimens for this study. Red circles indicate sampling sites of *Gammarus kyungsooki* sp. nov.; black circles, *G. sobaegensis*; black square, *G. odaensis*; black triangle, *G. soyoensis*; black diamond, *G. wangbangensis*.

*Gammarus* specimens were collected using hand nets from mountain streams at the sites indicated in Fig. 1 and Table 1. The collected organisms were immediately fixed in 95% ethanol and stored at -20℃. Body length measurements and specimen dissections were performed using a stereomicroscope (SZX12, Olympus, Japan), with body length measured along the dorsal margin from the base of antenna 1 to the base of the telson. All dissected appendages were mounted in glycerol on microscope slides and drawn using an optical microscope (DM2500, Leica, Germany), equipped with a drawing tube. The terminology of the setae in article 3 of the mandibular palp followed Cole (1980) and the description adhered to Ahn et al. (2022). All specimens were deposited at the National Institute of Biological Resources (NIBR), South Korea.

### Molecular analysis

Total genomic DNA was extracted from the left pleopod 1 of each specimen using a LaboPass Tissue Mini Kit (Cosmo Genetech, Seoul, South Korea). COI sequences were obtained using the primer set LCO1490-JJ (5′-CHACWAAYCATAAAGATATYGG-3′) and HCO2198-JJ (5′-AWACTTCVGGRTGVCCAAARAATCA-3′) (Astrin and Stüben 2010). PCR cycling conditions were as follows: initial denaturation at 94°C for 2 min; followed by 5 cycles of 30 s at 94°C, 30 s at 48°C, and 60 s at 72°C; then 30 cycles of 30 s at 94°C, 30 s at 53°C and 60 s at 72°C; and final extension at 72°C for 5 min. 28S sequences were obtained using the primer set 28F (5′-TTAGTAGGGGCGACCGAACAGGGAT-3′) and 28R (5′-GTCTTCGCCCCTATGCCCAACTGA-3′) (Hou et al. 2007). PCR cycling conditions were as follows: initial denaturation at 94°C for 2 min; followed by 35 cycles of 30 s at 94°C, 30 s at 52°C and 60 s at 72°C; and final extension at 72°C for 5 min.

COI and 28S sequences were aligned using Geneious 8.1.9 (Biomatters Ltd., Auckland, New Zealand). The aligned COI sequence was translated into amino acids to check for potential pseudogenes. Nucleotide sequence divergences were calculated using the uncorrected *p*-distance in MEGA X (Kumar et al. 2018). The details of the sequences obtained in this study and of those downloaded from GenBank are listed in Table 1.

To reconstruct the phylogeny of the genus *Gammarus*, the aligned COI and 28S sequences were concatenated into a single dataset. The best-fit nucleotide substitution model was selected using the Akaike Information Criterion (AIC) in jModelTest v.0.1.1 (Posada 2008). Phylogenetic analyses were conducted using Maximum Likelihood (ML) in RAxML v.8.2.9 (Stamatakis 2014) and Bayesian Inference (BI) in MrBayes v.3.2.1 (Ronquist et al. 2012), both under the GTR+I+G model. We used *Gammarus roeselii* as the outgroup.

## Taxon treatments

### Gammarus kyungsooki

Ahn & Min sp. nov.

488611C6-14B9-58DB-9A12-1250451003E9

69C8F63F-E014-470B-9766-DB3483D043B0

#### Materials

- **Type status:** Holotype. **Occurrence:** catalogNumber: NIBRIV0000929351; individualCount: 1; sex: male; lifeStage: adult; occurrenceID: 61CDDD6F-432A-5634-A699-733078101AFE; **Taxon:** scientificName: Gammarus kyungsooki; phylum: Arthropoda; class: Malacostraca; order: Amphipoda; family: Gammaridae; genus: Gammarus; specificEpithet: kyungsooki; taxonRank: species; scientificNameAuthorship: Ahn & Min; **Location:** country: South Korea; stateProvince: Gangwon-do; county: Cheorwon-gun; municipality: Sincheorwon-ri; verbatimLatitude: 38 08 41.82N; verbatimLongitude: 127 21 21.66E; verbatimCoordinateSystem: degrees minutes seconds; **Identification:** identifiedBy: Yong-Uk Ahn; **Event:** eventDate: 15 July 2021; **Record Level:** institutionCode: National Institute of Biological Resources (NIBR)
- **Type status:** Paratype. **Occurrence:** catalogNumber: NIBRIV0000931621; individualCount: 1; sex: female; lifeStage: adult; occurrenceID: 8F8677C3-F3A0-537A-904D-8AA516A648D7; **Taxon:** scientificName: Gammarus kyungsooki; phylum: Arthropoda; class: Malacostraca; order: Amphipoda; family: Gammaridae; genus: Gammarus; specificEpithet: kyungsooki; taxonRank: species; scientificNameAuthorship: Ahn & Min; **Location:** country: South Korea; stateProvince: Gangwon-do; county: Cheorwon-gun; municipality: Sincheorwon-ri; verbatimLatitude: 38 08 41.82N; verbatimLongitude: 127 21 21.66E; verbatimCoordinateSystem: degrees minutes seconds; **Identification:** identifiedBy: Yong-Uk Ahn; **Event:** eventDate: 15 July 2021; **Record Level:** institutionCode: National Institute of Biological Resources (NIBR)
- **Type status:** Paratype. **Occurrence:** catalogNumber: NIBRIV0000931622; individualCount: 1; sex: male; lifeStage: adult; occurrenceID: 961BB73C-9E9D-594A-9936-4D3221E30E6A; **Taxon:** scientificName: Gammarus kyungsooki; phylum: Arthropoda; class: Malacostraca; order: Amphipoda; family: Gammaridae; genus: Gammarus; specificEpithet: kyungsooki; taxonRank: species; scientificNameAuthorship: Ahn & Min; **Location:** country: South Korea; stateProvince: Gangwon-do; county: Cheorwon-gun; municipality: Sincheorwon-ri; verbatimLatitude: 38 08 41.82N; verbatimLongitude: 127 21 21.66E; verbatimCoordinateSystem: degrees minutes seconds; **Identification:** identifiedBy: Yong-Uk Ahn; **Event:** eventDate: 15 July 2021; **Record Level:** institutionCode: National Institute of Biological Resources (NIBR)
- **Type status:** Paratype. **Occurrence:** catalogNumber: NIBRIV0000931623; individualCount: 1; sex: male; lifeStage: adult; occurrenceID: 961BB73C-9E9D-594A-9936-4D3221E30E6A; **Taxon:** scientificName: Gammarus kyungsooki; phylum: Arthropoda; class: Malacostraca; order: Amphipoda; family: Gammaridae; genus: Gammarus; specificEpithet: kyungsooki; taxonRank: species; scientificNameAuthorship: Ahn & Min; **Location:** country: South Korea; stateProvince: Gangwon-do; county: Cheorwon-gun; municipality: Sincheorwon-ri; verbatimLatitude: 38 08 41.82N; verbatimLongitude: 127 21 21.66E; verbatimCoordinateSystem: degrees minutes seconds; **Identification:** identifiedBy: Yong-Uk Ahn; **Event:** eventDate: 15 July 2021; **Record Level:** institutionCode: National Institute of Biological Resources (NIBR)
- **Type status:** Paratype. **Occurrence:** catalogNumber: NIBRIV0000931624; individualCount: 7; sex: 4 males and 3 females; lifeStage: adult; occurrenceID: B8771A33-2559-55BD-BFD0-CA0770E4CBE9; **Taxon:** scientificName: Gammarus kyungsooki; phylum: Arthropoda; class: Malacostraca; order: Amphipoda; family: Gammaridae; genus: Gammarus; specificEpithet: kyungsooki; taxonRank: species; scientificNameAuthorship: Ahn & Min; **Location:** country: South Korea; stateProvince: Gangwon-do; county: Cheorwon-gun; municipality: Sincheorwon-ri; verbatimLatitude: 38 08 41.82N; verbatimLongitude: 127 21 21.66E; verbatimCoordinateSystem: degrees minutes seconds; **Identification:** identifiedBy: Yong-Uk Ahn; **Event:** eventDate: 15 July 2021; **Record Level:** institutionCode: National Institute of Biological Resources (NIBR)
- **Type status:** Paratype. **Occurrence:** catalogNumber: NIBRIV0000931625; individualCount: 1; sex: male; lifeStage: adult; occurrenceID: 5AA5CCDF-5798-54DE-A25B-6842DC019E3A; **Taxon:** scientificName: Gammarus kyungsooki; phylum: Arthropoda; class: Malacostraca; order: Amphipoda; family: Gammaridae; genus: Gammarus; specificEpithet: kyungsooki; taxonRank: species; scientificNameAuthorship: Ahn & Min; **Location:** country: South Korea; stateProvince: Gangwon-do; county: Hwacheon-gun; municipality: Bongo-ri; verbatimLatitude: 38 11 24.48N; verbatimLongitude: 127 34 53.94E; verbatimCoordinateSystem: degrees minutes seconds; **Identification:** identifiedBy: Yong-Uk Ahn; **Event:** eventDate: 16 July 2021; **Record Level:** institutionCode: National Institute of Biological Resources (NIBR)
- **Type status:** Paratype. **Occurrence:** catalogNumber: NIBRIV0000931626; individualCount: 1; sex: male; lifeStage: adult; occurrenceID: 6038F215-7A5B-5318-870F-34B1782F268F; **Taxon:** scientificName: Gammarus kyungsooki; phylum: Arthropoda; class: Malacostraca; order: Amphipoda; family: Gammaridae; genus: Gammarus; specificEpithet: kyungsooki; taxonRank: species; scientificNameAuthorship: Ahn & Min; **Location:** country: South Korea; stateProvince: Gangwon-do; county: Hwacheon-gun; municipality: Bongo-ri; verbatimLatitude: 38 11 24.48N; verbatimLongitude: 127 34 53.94E; verbatimCoordinateSystem: degrees minutes seconds; **Identification:** identifiedBy: Yong-Uk Ahn; **Event:** eventDate: 16 July 2021; **Record Level:** institutionCode: National Institute of Biological Resources (NIBR)
- **Type status:** Paratype. **Occurrence:** catalogNumber: NIBRIV0000931627; individualCount: 1; sex: female; lifeStage: adult; occurrenceID: DFEC76CB-F226-549F-B526-7EFD12EF45D5; **Taxon:** scientificName: Gammarus kyungsooki; phylum: Arthropoda; class: Malacostraca; order: Amphipoda; family: Gammaridae; genus: Gammarus; specificEpithet: kyungsooki; taxonRank: species; scientificNameAuthorship: Ahn & Min; **Location:** country: South Korea; stateProvince: Gangwon-do; county: Hwacheon-gun; municipality: Bongo-ri; verbatimLatitude: 38 11 24.48N; verbatimLongitude: 127 34 53.94E; verbatimCoordinateSystem: degrees minutes seconds; **Identification:** identifiedBy: Yong-Uk Ahn; **Event:** eventDate: 16 July 2021; **Record Level:** institutionCode: National Institute of Biological Resources (NIBR)
- **Type status:** Paratype. **Occurrence:** catalogNumber: NIBRIV0000931628; individualCount: 4; sex: 3 males and 1 female; lifeStage: adult; occurrenceID: DFEC76CB-F226-549F-B526-7EFD12EF45D5; **Taxon:** scientificName: Gammarus kyungsooki; phylum: Arthropoda; class: Malacostraca; order: Amphipoda; family: Gammaridae; genus: Gammarus; specificEpithet: kyungsooki; taxonRank: species; scientificNameAuthorship: Ahn & Min; **Location:** country: South Korea; stateProvince: Gangwon-do; county: Hwacheon-gun; municipality: Bongo-ri; verbatimLatitude: 38 11 24.48N; verbatimLongitude: 127 34 53.94E; verbatimCoordinateSystem: degrees minutes seconds; **Identification:** identifiedBy: Yong-Uk Ahn; **Event:** eventDate: 16 July 2021; **Record Level:** institutionCode: National Institute of Biological Resources (NIBR)
- **Type status:** Paratype. **Occurrence:** catalogNumber: NIBRIV0000931629; individualCount: 4; sex: 3 males and 2 females; lifeStage: adult; occurrenceID: DFEC76CB-F226-549F-B526-7EFD12EF45D5; **Taxon:** scientificName: Gammarus kyungsooki; phylum: Arthropoda; class: Malacostraca; order: Amphipoda; family: Gammaridae; genus: Gammarus; specificEpithet: kyungsooki; taxonRank: species; scientificNameAuthorship: Ahn & Min; **Location:** country: South Korea; stateProvince: Gangwon-do; county: Cheorwon-gun; municipality: Munhye-ri; verbatimLatitude: 38 11 49.33N; verbatimLongitude: 127 23 32.64E; verbatimCoordinateSystem: degrees minutes seconds; **Identification:** identifiedBy: Yong-Uk Ahn; **Event:** eventDate: 22 October 2025; **Record Level:** institutionCode: National Institute of Biological Resources (NIBR)
- **Type status:** Paratype. **Occurrence:** catalogNumber: NIBRIV0000931630; individualCount: 3; sex: 2 males and 1female; lifeStage: adult; occurrenceID: DFEC76CB-F226-549F-B526-7EFD12EF45D5; **Taxon:** scientificName: Gammarus kyungsooki; phylum: Arthropoda; class: Malacostraca; order: Amphipoda; family: Gammaridae; genus: Gammarus; specificEpithet: kyungsooki; taxonRank: species; scientificNameAuthorship: Ahn & Min; **Location:** country: South Korea; stateProvince: Gangwon-do; county: Cheorwon-gun; municipality: Jamgok-ri; verbatimLatitude: 38 08 43.41N; verbatimLongitude: 127 27 38.72E; verbatimCoordinateSystem: degrees minutes seconds; **Identification:** identifiedBy: Yong-Uk Ahn; **Event:** eventDate: 22 October 2025; **Record Level:** institutionCode: National Institute of Biological Resources (NIBR)

#### Description

**Figure 2.**
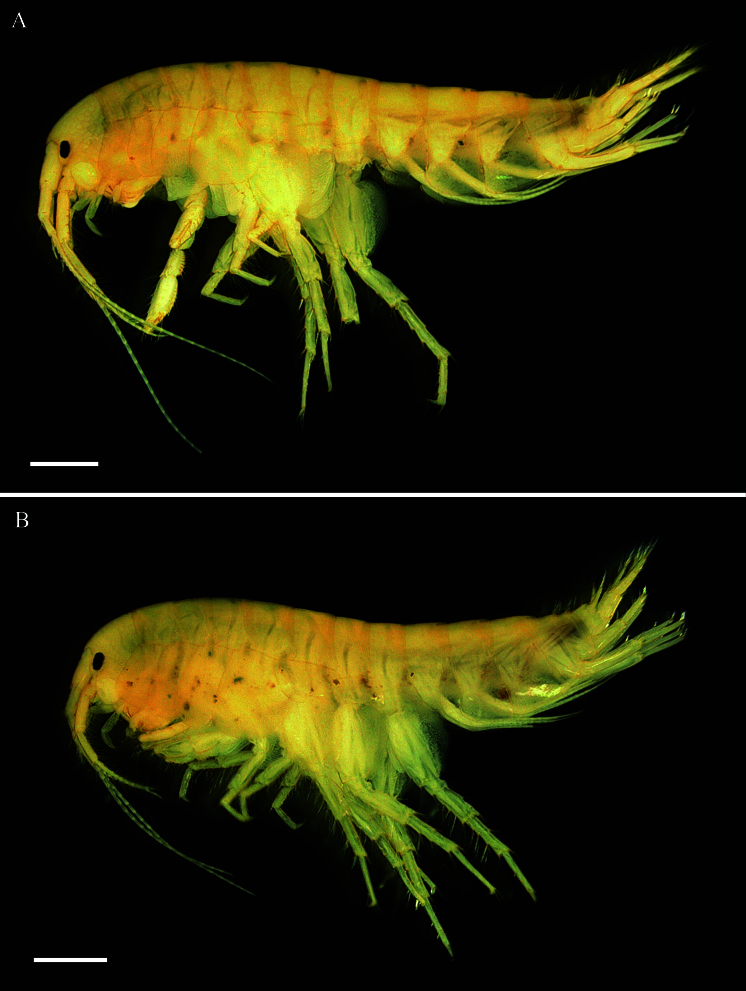
Habitus of *Gammarus kyungsooki* sp. nov. **A** male, holotype (QUQUIV0000001642); **B** female, paratype (NIBR00001). Scale bar: 1 mm.

**Description of male, based on holotype** (Fig. 2A, QUQUIV0000001642).

**Figure 3.**
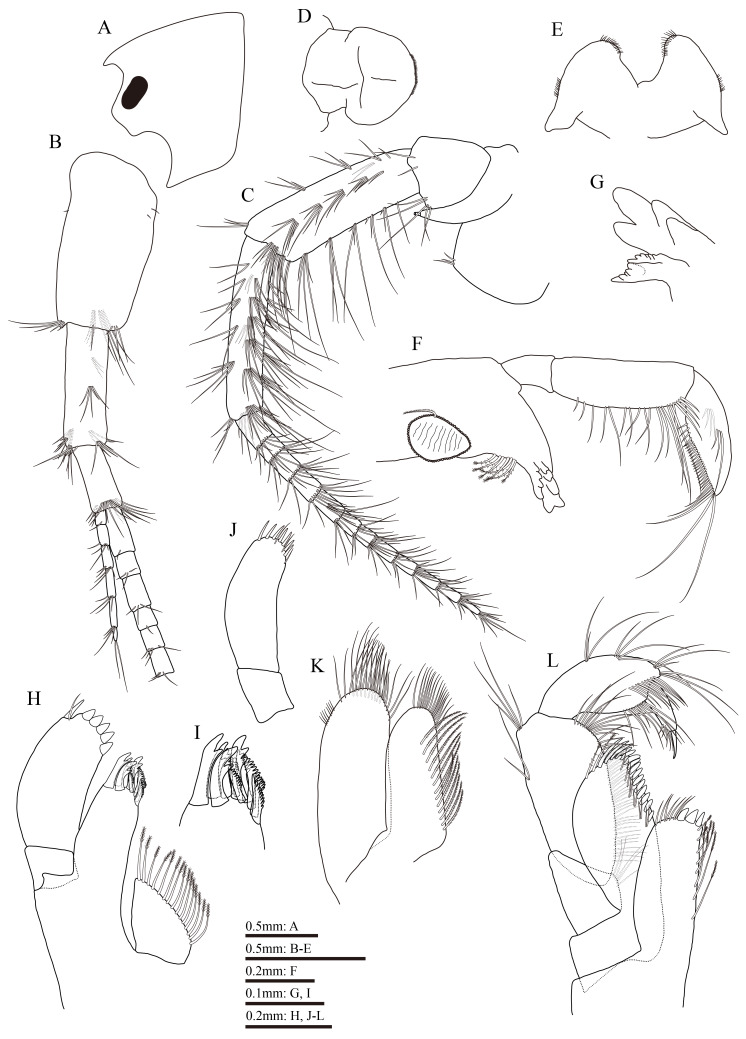
*Gammarus kyungsooki* sp. nov., male, holotype (QUQUIV0000001642). **A** head; **B** antenna 1, omitted from main flagellar article 7; **C** antenna 2; **D** upper lip; **E** lower lip; **F** left mandible; **G** incisor and lacinia mobilis of right mandible; **J** right maxilla 1; **I** outer plate of right maxilla 1; **J** palp of left maxilla 1; **K** maxilla 2; **L** maxilliped.

Body length 8.1 mm. Head (Fig. 3A): eyes reniform, rostrum short, inferior antennal sinus deep and rounded.

Antenna 1 (Fig. 3B): approximately 0.6 times as long as body length; peduncular articles 1–3 in length ratio 1: 0.7: 0.4, with distal setae clusters on each peduncular article; main flagellum 23-articulated; accessory flagellum 6-articulated; both flagella with short distal setae.

Antenna 2 (Fig. 3C): peduncular article 1 with 3 short distal setae; gland cone prominent and tapering distally, reaching tip of peduncular article 3; anterior, posterior and interior margins of peduncular article 4 with four, seven and six clusters of long setae, respectively, length of longest seta on posterior margin approximately 2.0 times the width of peduncular article 4; peduncular article 5 almost equal length to article 4, anterior, posterior and interior margins with six, seven and six clusters of long setae, respectively; length of longest seta on posterior margin 2.8 times as long as width of peduncular article 5; flagellum 12-articulated, calceoli absent.

Upper lip (Fig. 3D): rounded, numerous minute setules on ventral margin.

Lower lip (Fig. 3E): inner lobes indistinct, outer lobes broad and covered with thin setules.

Mandible (Fig. 3F and G): incisor of left and right mandibles with five and four teeth, respectively; left lacinia mobilis with four teeth, right lacinia mobilis bifurcate, with small teeth; molar triturative, with one plumose seta; palp 3-articulated in length ratio 1.0:2.6:2.3, article 1 unarmed, article 2 with 20 marginal setae, article 3 bearing three B-setae on inner surface, four A-setae on outer surface, 23 D-setae and 5 E-setae.

Maxilla 1 (Fig. 3H–J): medial margin of inner plate with 15 plumose setae; outer plate bearing 11 serrated spines apically; palp two-articulated and asymmetrical, right palp shorter and stouter than left palp, article 2 of right palp with five stout spines, one slender spine and one seta apically; article 2 of left palp with seven slender spines and three setae apically.

Maxilla 2 (Fig. 3K): inner plate with 13 plumose setae in an oblique row; outer plate broader than inner plate; both plates with numerous long apical setae.

Maxilliped (Fig. 3L): inner plate with three apical stout spines, several plumose setae on lateral margin; outer plate bearing a row of blade-like spines and two plumose setae; palp 4-articulated, article 1 unarmed, inner margin of article 2 with numerous setae, article 3 curved, bearing numerous setae on inner margin and a row of subapical setae; article 4 hooked, with four setae on hinge of unguis.

**Figure 4.**
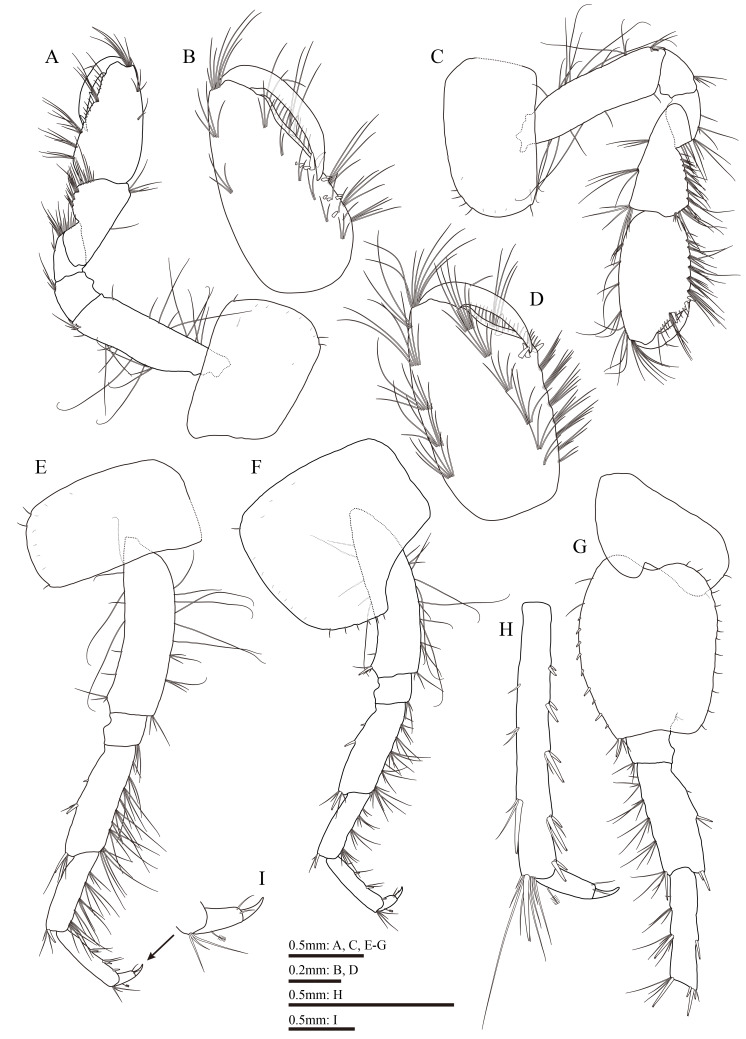
*Gammarus kyungsooki* sp. nov., male, holotype (QUQUIV0000001642). **A** gnathopod 1; **B** propodus and dactylus of gnathopod 1; **C** gnathopod 2; **D** propodus and dactylus of gnathopod 2; **E** pereopod 3; **F** pereopod 4; **G** coxal plate to carpus of left pereopod 5; **H** propodus to dactylus of right pereopod 5.

Gnathopod 1 (Fig. 4A and B): coxal plate with two setae on anteroventral corner and one seta on posteroventral corner; basis with setae of various lengths on anterior and posterior margins; length of carpus 1.7 times the width, approximately 0.75 times the length of propodus, bearing three clusters of setae along ventral margin; propodus pyriform, palmar margin oblique, with one medial palmar spine and 12 short spines on posterior margin; dactylus with one seta on outer margin, exceeding half of propodus.

Gnathopod 2 (Fig. 4C and D): coxal plate with four setae on anteroventral corner and one seta on posteroventral corner; basis with setae of various lengths on anterior and posterior margins; length of carpus 1.9 times the width, approximately 0.8 times the length of propodus, with six clusters of setae on ventral margin; propodus subrectangular, palmar margin concave, with one medial palmar spine and four spines on posterodistal corner; dactylus curved beyond the palmar margin, with one seta on outer margin.

Pereopod 3 (Fig. 4E): coxal plate bearing two setae on anteroventral corner and one seta on posteroventral corner; anterior and posterior margins of basis with setae of various lengths; merus more elongate than that of pereopod 4, with two spines on anterior margin and seven clusters of long straight setae on posterior margin, longest setae on posterior margin reaching approximately 2.0 times the width of merus; carpus with four clusters of long straight setae along posterior margin, anterior margin unarmed; propodus with three spines accompanied by setae on posterior margin; dactylus with one penicillate seta on anterior margin and two setae on hinge of unguis.

Pereopod 4 (Fig. 4F): shorter than pereopod 3; coxal plate excavated posterodistally, with one seta on anteroventral corner and six setae on posterior margin; basis similar to that of pereopod 3, except one spine on anterodistal corner; merus with four clusters and two single straight setae on posterior margin, longest setae of these longer than the width of merus; carpus with three clusters of straight setae on posterior margin; posterior margin of propodus with spines and setae; dactylus similar to that of pereopod 3.

Pereopod 5 (Fig. 4G and H): coxal plate bilobed, bearing two setae on posterior margin of posterior lobe; basis with two setae and a row of nine spines on anterior margin, anterodistal corner with single spine accompanied by setae, posterior margin with a row of 13 short setae, posterodistal lobe developed; merus with four clusters of setae on anterior margin, a spine accompanied by seta on posterior margin; carpus with two clusters of setae and one spine on anterior margin, one spine on posterior margin; propodus with four pairs of spines on anterior margin; dactylus with one penicillate seta on posterior margin and two setae on hinge of unguis.

**Figure 5.**
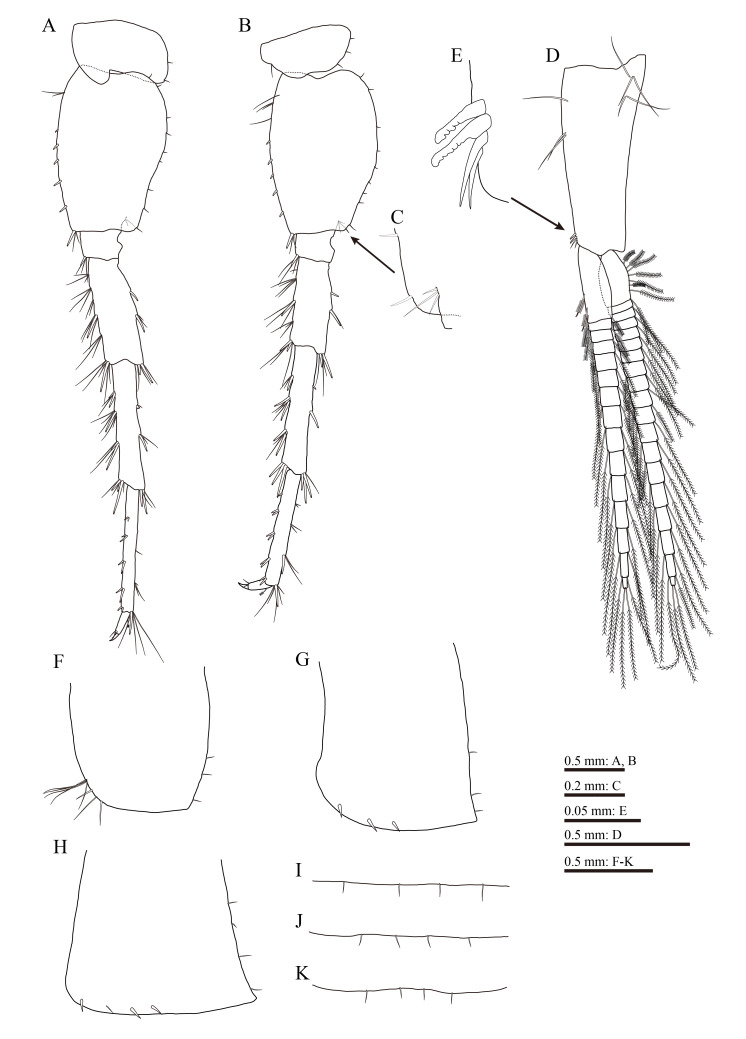
*Gammarus kyungsooki* sp. nov., male, holotype (QUQUIV0000001642). **A** pereopod 6; **B** pereopod 7; **C** inner surface near posterodistal corner of pereopod 7 basis; **D** pleopod 1; **E** inner distal corner of peduncle in pleopod 1; **F–H**, epimeral plates 1–3, respectively; **I–K**, pleonites 1–3, respectively.

Pereopod 6 (Fig. 5A): longer than pereopod 5, coxal plate bilobed, with two short setae on posterior margin of posterior lobe; basis with two setae and a row of five spines on anterior margin, single spine accompanied by setae on anterodistal corner, and a row of nine short setae along posterior margin, posterodistal lobe not developed; merus with five clusters of setae and one spine on anterior margin, two spines on posterior margin; carpus with three groups of spines accompanied by setae on anterior margin; propodus with four groups of spines on anterior margin; dactylus similar to that of pereopod 5.

Pereopod 7 (Fig. 5B and C): coxal plate shallowly concave ventrally, bearing one seta on anterior margin and three setae on posterior margin; basis with four setae and a row of five spines on anterior margin, single spine accompanied by setae on anterodistal corner and a row of nine short setae on posterior margin, posterodistal lobe not developed, inner surface near posterodistal corner with three setae; merus with four clusters of setae and one spine on anterior margin, two spines on posterior margin; carpus with three groups of spines accompanied by setae on anterior margin; propodus with four pairs of spines on anterior margin; dactylus similar to those of pereopod 5 and 6.

Coxal gills present on gnathopod 2 and pereopods 3–7.

Pleopods (Fig. 5D and E): peduncle with several long marginal setae, two retinacula accompanied by two setae on inner distal corner; both rami almost equal in length, fringed with plumose setae.

Epimeral plates 1–3 (Fig. 5F–H): plate 1 with eight setae on anteroventral margin and three short setae on posterior margin; plate 2 with three spines and three short setae on ventral and posterior margins, respectively; plate 3 with three spines and one seta on ventral margin and four short setae on posterior margin.

Pleonites 1–3 (Fig. 5I–M): each plate with four setae on posterodorsal margin, respectively.

**Figure 6.**
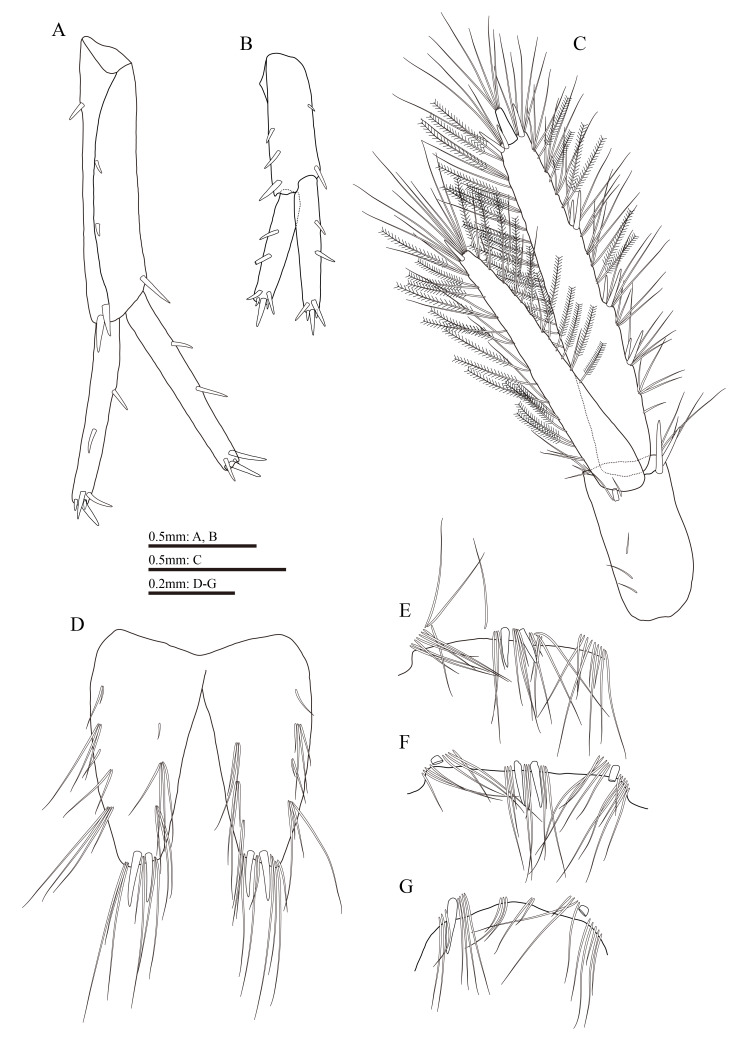
*Gammarus kyungsooki* sp. nov., male, holotype (QUQUIV0000001642). **A** uropod 1; **B** uropod 2; **C** uropod 3; **D** telson; **E–G** urosomites 1–3, respectively.

Uropod 1 (Fig. 6A): peduncle with one basofacial spine, two spines on outer margin, two spines and one spine on outer and inner distal corners, respectively; inner ramus 0.7 times the length of peduncle, with two spines on inner margin; the length of outer ramus as long as inner ramus, with one spine on inner and outer margins each; both rami with five unequal distal spines.

Uropod 2 (Fig. 6B): peduncle with one and two spines on inner and outer margins, respectively, one spine on each distal corner; inner ramus subequal to the length of peduncle and 1.2 times as long as outer ramus, with two spines on inner margin; outer ramus with two spines on outer margin; both rami with five unequal distal spines.

Uropod 3 (Fig. 6C): peduncle with several distal spines accompanied by setae; inner ramus 1.8 times the length of peduncle and 0.74 times as long as outer ramus, both margins densely set with plumose and simple setae; outer ramus 2-articulate, proximal article with three pairs of spines on outer margin and three spines on distal end, both margins densely set with plumose and simple setae; length of setae on outer margin longer than width of proximal article, terminal article prominent, slightly longer than adjacent spines.

Telson (Fig. 6D): deeply cleft nearly to the base, almost equal in length and width, each lobe with two distal spines and accompanied by long setae, left lobe with four clusters of setae and one single seta on outer margin, two clusters of setae and one single seta on inner margin, right lobe with three clusters of setae and one single seta on outer margin, three clusters of setae on inner margin.

Urosomites 1–3 (Fig. 6E–G): dorsally flat, urosomite 1 with a cluster of setae without spine on each side and two spines accompanied by a cluster of setae on dorsal margin; urosomite 2 with 1–2–1 spine formula accompanied by a cluster of setae on dorsal margin; urosomite 3 with one spine accompanied by a cluster of setae on each side and two clusters of setae on dorsal margin.

**Description of female, based on paratype (Fig. 2B, NIBRIV00001)**

**Figure 7.**
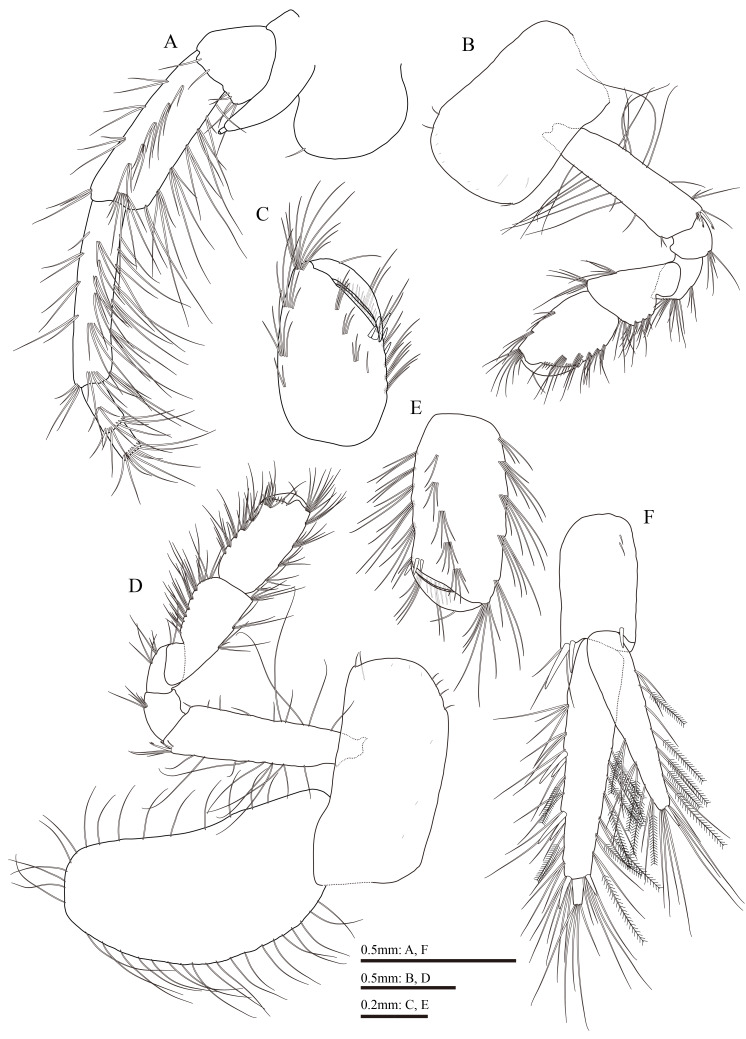
*Gammarus kyungsooki* sp. nov., female, paratype (NIBR00001). **A** antenna 2, omitted from flagella article 3; **B** gnathopod 1; **C** propodus and dactylus of gnathopod 1; **D** gnathopod 2; **E** propodus and dactylus of gnathopod 2; **F** uropod 3.

Body length 7.6 mm. General appearance similar to male. Differences from male as follows:

Antenna 2 (Fig. 7A): setae of peduncular article 4, 5 longer than those of male, longest seta implanted in posterior margin 2.4 and 2.9 times as long as widths of peduncular article 4 and 5, respectively.

Gnathopod 1 (Fig. 7B and C): carpus broader than that of male, length 1.3 times the width; palmar margin of propodus not as oblique as that of male, with five spines on posterodistal corner, medial palmar spine absent; dactylus not reaching half of propodus.

Gnathopod 2 (Fig. 7D and E): carpus more elongate than that of male, length of carpus approximately 2.1 times the width, 1.1 times as long propodus; palmar margin of propodus with three spines on posterodistal corner, medial palmar spine absent.

Oostegites: present on gnathopod 2 (Fig. 7D) and pereopods 3-5, with numerous marginal setae.

Uropod 3 (Fig. 7F): both rami shorter than those of male, inner ramus 1.4 times the length of peduncle and 0.7 times the length of outer ramus.

#### Diagnosis

The posterior margins of peduncular article 4 and 5 of antenna 2 with 6–7 and 7–8 clusters of long setae, respectively, flagellum without calceoli; pereopod 3 with long straight setae on posterior margins of merus to carpus; inner ramus of uropod 3 approximately 0.7 times the length of outer ramus, urosomite 1 with a 0–2–0 dorsal spine formula; outer margin of outer ramus with plumose setae; telson with three or more clusters of setae on outer margin of each lobe.

#### Etymology

The specific name is in honour of Professor Emerita Kyung Sook Lee for her significant contributions to the taxonomy of freshwater amphipods in Korea, especially the genus *Gammarus*.

#### Distribution

The new species was collected under decomposing leaves from the upper Hantan River zone, South Korea (Fig. 1).

#### Molecular data

**Figure 8.**
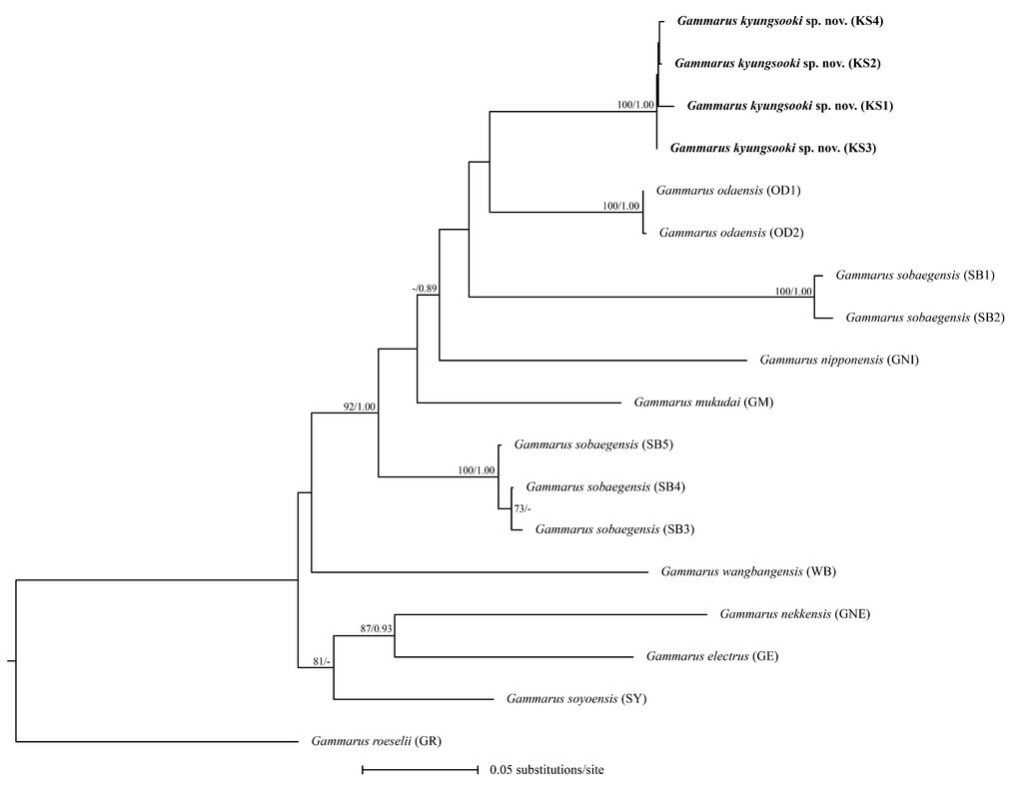
Maximum-Likelihood tree of the COI + 28S rRNA gene sequences. Numbers above the branches are ML bootstraps/Bayesian posterior probabilities. Bootstraps and Bayesian posterior probabilities are shown when over 70% and 0.85, respectively.

COI (GenBank accession numbers: XX000001–XX000004) and 28S (GenBank accession numbers: XY000001–XY000004) gene sequences were obtained from four specimens of the new species. Maximum intraspecific divergence was 1.3% for COI and 0.5% for 28S (Table 2). Interspecific divergence between the new species and related species ranged from 18.8% to 25.2% for COI and from 1.7% to 5.1% for 28S (Table 2). The ML and BI trees shared the same topology and the new species formed a well-supported monophyletic clade with high support values (Fig. 8).

#### Remarks

Thus far, 12 endemic *Gammarus* species have been reported from South Korea: *G. baengnyeongensis* Kwon, Kim, Heo & Kim, 2020; *G. gageoensis* Kim, Lee & Min, 2010; *G. galgosensis* Lee & Kim, 1980; *G. hoonsooi* Lee, 1986; *G. kyonggiensis* Lee & Seo, 1990; *G. longisaeta* Lee & Seo, 1992; *G. odaensis* Lee & Kim, 1980; *G. sobaegensis* Uéno, 1966; *G. somaemulensis* Ahn, Lee & Min, 2022; *G. soyoensis* Lee & Kim, 1980; *G. wangbangensis* Lee & Seo, 1992; and *G. zeongogensis* Lee & Kim, 1980 (Uéno 1966, Lee and Kim 1980, Lee 1986, Lee and Seo 1990, Lee and Seo 1992, Kim et al. 2010, Kwon et al. 2020, Ahn et al. 2022). Amongst them, four species (*G. sobaegensis*, *G. wangbangensis*, *G. soyoensis* and *G. odaensis*) resemble *Gammarus kyungsooki* **sp. nov.** in the following features: antenna 2 with long setae on the peduncle and lacking calceoli; pereopods 3 and 4 with straight setae on posterior margins; bases of pereopods 5–7 with short setae on posterior margins; and the length ratio of inner and outer rami of uropod 3 approximately 0.7 or more. However, the new species can be distinguished by the combination of the following features: posterior margin of peduncular article 4 of antenna 2 with 6–7 clusters of setae; straight setae of pereopod 3 are long; spine formula of urosomite 1 is a 0–2–0; presence of plumose setae on outer margin of outer ramus in uropod 3; telson with three or more clusters of lateral setae (Table 3).

The interspecific divergences calculated in this study are comparable to, or even higher than, those reported in previous studies on freshwater *Gammarus*. Hou and Li (2010) reported interspecific divergences amongst Chinese *Gammarus* species of 14.8%–18.8% and 3.27%–3.37% for COI and 28S, respectively. Similarly, Özbek et al. (2023) reported divergences of 12.2% for COI and 0.6% for 28S between two morphologically similar species, *G. tumaf* and *G. baysali*. Therefore, the COI and 28S divergences between *G. kyungsooki* and its related species (18.8%–25.2% and 1.7%–5.1%, respectively) support its distinct species status.

The *G. sobaegensis* specimens collected from the Hantan River region showed remarkably high intraspecific divergence (maximum 22.1% for COI and 5.3% for 28S) and formed two distinct lineages (SB1+SB2 and SB3+SB4+SB5) (Fig. 8, Table 3). This species has a broad distribution across South Korea (Lee and Kim 1980) and the phylogenetic analyses by Hu et al. (2021) indicate the presence of additional genetic lineages. *G. sobaegensis* may comprise multiple cryptic species and additional integrative taxonomic studies are required to confirm its species boundaries.

#### New Korean name

Teol-kko-ri-yeop-sae-u (털꼬리옆새우)

## Supplementary Material

XML Treatment for Gammarus kyungsooki

## Figures and Tables

**Table 1.** Information of *Gammarus* specimens and GenBank accession numbers used in this study. Sampling codes correspond to those in Fig. 1.

Code	Species	Locality	Latitude, longitude	COI	28S
KS1	*G. kyungsooki* sp. nov.	Sincheorwon-ri, Galmal-eup, Cheorwon-gun, South Korea	38°08'41.82"N, 127°21'21.66"E	PX879554	PX898325
KS2	*G. kyungsooki* sp. nov.	Munhye-ri, Galmal-eup, Cheorwon-gun, South Korea	38°11'49.33"N, 127°23'32.64"E	PX890090	PX898326
KS3	*G. kyungsooki* sp. nov.	Jamgok-ri, Geunnam-myeon, Cheorwon-gun, South Korea	38°08'43.41"N, 127°27'38.72"E	PX890091	PX898327
KS4	*G. kyungsooki* sp. nov.	Bong-o-ri, Sangseo-myeon, Hwacheon-gun, South Korea	38°11'24.48"N, 127°34'53.94"E	PX890092	PX898328
SB1	*G. sobaegensis*	Maji-ri, Juksung-myeon, Paju-si, South Korea	37°56'43.32"N, 126°55'21.72"E	PX890096	PX898329
SB2	*G. sobaegensis*	Choseong-ri, Cheongsan-myeon, Yeoncheon-gun, South Korea	37°59'20.27"N, 127°05'25.05"E	PX890097	PX898330
SB3	*G. sobaegensis*	Gomun-ri, Yeoncheon-eup, Yeoncheon-gun, South Korea	38°04'37.98"N, 127°08'35.88"E	PX890098	PX898331
SB4	*G. sobaegensis*	Sangno-ri, Dongsong-eup, Cheorwon-gun, South Korea	38°09'34.14"N, 127°11'32.10"E	PX890099	PX898332
SB5	*G. sobaegensis*	Sanjeong-ri, Yeongbuk-myeon, Pocheon-si, South Korea	38°03'56.22"N, 127°19'34.80"E	PX890100	PX898333
OD1	*G. odaensis*	Sineup-ri, Hwacheon-eup, Hwacheon-gun, South Korea	38°08'51.73"N, 127°42'35.35"E	PX892994	PX900189
OD2	*G. odaensis*	Cheonmi-ri, Bangsan-myeon, Yanggu-gun, South Korea	38°13'47.69"N, 127°52'38.18"E	PX892995	PX900190
SY	*G. soyoensis*	Sangbongam-dong, Dongducheon-si, South Korea	37°56'39.50"N, 127°05'17.00"E	ON980559	PX898334
WB	*G. wangbangensis*	Kiji-ri, Sinbuk-myeon, Pocheon-si, South Korea	37°54'58.00"N, 127°14'09.40"E	ON980560	PX898335
GM	G. mukudai	Katsumoto, Iki, Nagasaki, Japan	33°49'38.80"N, 129°42'35.10"E	AB893343	AB893233
GNI	G. nipponensis	Yao, Osaka, Japan	unknown	AB893314	AB893204
GE	G. electrus	Haidian, Beijing, China	39°53'59.90"N, 116°24'00.00"E	EF570305	EF582951
GNE	G. nekkensis	Heilongjiang Province, China	46°30'36.00"N, 133°16'48.00"E	OK210030	OK255410
GR	G. roeselii	Coulange, France	37°54'58.00"N, 127°14'09.40"E	JF965953	JF965778

**Table 2.** A matrix of the uncorrected *p*-distance values amongst *G. kyungsooki* sp. nov. and related species. The codes before the species names indicate the sampling codes corresponding to those in Table 1. COI-based distances are presented below the diagonal and 28S-based distances above the diagonal.

	**(Genus *Gammarus*)**	**1**	**2**	**3**	**4**	**5**	**6**	**7**	**8**	**9**	**10**	**11**	**12**	**13**
**1**	*G. kyungsooki* sp. nov. (KS1)		0.005	0.005	0.005	0.049	0.048	0.022	0.021	0.022	0.019	0.019	0.029	0.040
**2**	*G. kyungsooki* sp. nov. (KS2)	0.012		0.000	0.000	0.051	0.050	0.020	0.019	0.020	0.017	0.017	0.027	0.036
**3**	*G. kyungsooki* sp. nov. (KS3)	0.010	0.005		0.000	0.051	0.050	0.020	0.019	0.020	0.017	0.017	0.027	0.036
**4**	*G. kyungsooki* sp. nov. (KS4)	0.013	0.007	0.007		0.051	0.050	0.020	0.019	0.020	0.017	0.017	0.027	0.036
**5**	*G. sobaegensis* (SB1)	0.201	0.204	0.206	0.206		0.003	0.052	0.053	0.052	0.052	0.052	0.059	0.069
**6**	*G. sobaegensis* (SB2)	0.204	0.208	0.209	0.209	0.026		0.049	0.050	0.049	0.049	0.049	0.056	0.068
**7**	*G. sobaegensis* (SB3)	0.211	0.216	0.213	0.213	0.213	0.219		0.001	0.000	0.011	0.011	0.019	0.032
**8**	*G. sobaegensis* (SB4)	0.213	0.217	0.214	0.214	0.216	0.221	0.012		0.001	0.010	0.010	0.020	0.033
**9**	*G. sobaegensis* (SB5)	0.208	0.213	0.209	0.209	0.214	0.221	0.028	0.016		0.011	0.011	0.019	0.032
**10**	*G. odaensis* (OD1)	0.188	0.189	0.186	0.191	0.201	0.204	0.221	0.219	0.217		0.000	0.022	0.036
**11**	*G. odaensis* (OD2)	0.189	0.191	0.188	0.193	0.203	0.206	0.219	0.217	0.219	0.003		0.022	0.036
**12**	*G. soyoensis* (SY)	0.247	0.252	0.249	0.250	0.226	0.234	0.213	0.213	0.213	0.219	0.217		0.026
**13**	*G. wangbangensis* (WB)	0.232	0.234	0.234	0.232	0.250	0.255	0.244	0.242	0.242	0.260	0.264	0.245	

**Table 3.** Comparison of morphological characteristics amongst *Gammarus kyungsooki* sp. nov. and related species.

**Character (male)**	***G. kyungsooki*** **sp. nov.**	***G. sobaegensis***	***G. wangbangensis***	***G. soyoensis***	***G. odaensis***
**Antenna 2**					
Peduncle 4 posterior setae clusters	6–7	6–8	3–4	7–8	7–9
**Gnathopod 1**					
Medial palmar spine	Present	Present	Present	Absent	Present
**Gnathopod 2**					
Medial palmar spine	Present	Present	Present	Absent	Present
**Pereopod 3**					
Setae on posterior margin	Long straight setae	Long straight setae	Long straight setae	Long straight setae	Short straight setae
**Pereopod 7**					
Posterodistal corner of basis	Setae	Spine	Setae	Setae	Setae
Setae on anterior margin	Present	Present	Present	Present	Absent (spines only)
**Uropod 3**					
Plumose setae on outer margin of outer ramus	Present	Absent	Present	Present	Absent
Outer marginal setae of outer ramus	Long	Long	Long	Short	Short
**Urosomite 1**					
Dorsal spine formula	0–2–0	1–2–1 or 2–2–2	1–2–1	1–2–1	1–2–1
**Telson**					
Lateral setae clusters	3–4	1–2	2	2	2
**Reference**	This study	Uéno (1966)	Lee & Seo (1990)	Lee & Kim (1980)	Lee & Kim (1980)
